# Acetoguanamine *N*,*N*-dimethyl­formamide solvate

**DOI:** 10.1107/S1600536808023842

**Published:** 2008-08-06

**Authors:** Gustavo Portalone

**Affiliations:** a Chemistry Department, "Sapienza" University of Rome, P. le A. Moro, 5, I-00185 Rome, Italy

## Abstract

The structure of acetoguanamine (or 2,4-diamino-6-methyl-1,3,5-triazine) has been determined as the *N*,*N*-dimethyl­formamide solvate, C_4_H_7_N_5_·C_3_H_7_NO. The mol­ecular components are associated in the crystal structure to form ribbons stabilized by three N—H⋯N and one N—H⋯O hydrogen bonds which involve NH groups as donors and the N atoms of the heterocyclic ring and the carbonyl O atom of the solvent as acceptors.

## Related literature

For related literature, see: Portalone & Colapietro (2007*a* ▶). For a general approach to the use of multiple-hydrogen-bonding DNA/RNA nucleobases as potential supra­molecular reagents, see: Portalone *et al.* (1999 ▶); Portalone & Colapietro (2007*a* ▶,*b* ▶ and references therein). For the computation of ring patterns formed by hydrogen bonds in crystal structures, see: Etter *et al.* (1990 ▶); Bernstein *et al.* (1995 ▶); Motherwell *et al.* (1999 ▶).
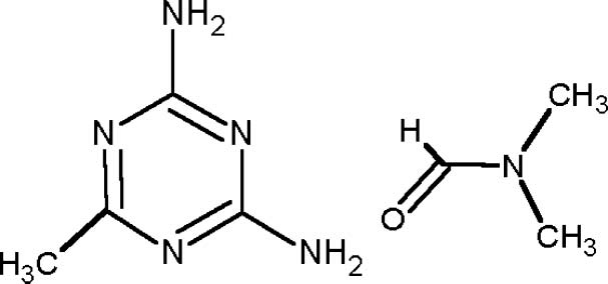


## Experimental

### 

#### Crystal data

C_4_H_7_N_5_·C_3_H_7_NO
*M* *_r_* = 198.24
Orthorhombic, 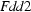

*a* = 25.548 (2) Å
*b* = 23.0626 (19) Å
*c* = 7.2689 (9) Å
*V* = 4282.8 (7) Å^3^
*Z* = 16
Mo *K*α radiation
μ = 0.09 mm^−1^
*T* = 298 (2) K
0.15 × 0.14 × 0.14 mm

#### Data collection

Oxford Diffraction Xcalibur S CCD diffractometer
Absorption correction: multi-scan (*CrysAlis RED*; Oxford Diffraction, 2006 ▶) *T*_min_ = 0.985, *T*_max_ = 0.990
27177 measured reflections
1127 independent reflections
698 reflections with *I* > 2σ(*I*)
*R*_int_ = 0.064

#### Refinement

*R*[*F* ^2^ > 2σ(*F* ^2^)] = 0.047
*wR*(*F* ^2^) = 0.127
*S* = 0.91
1127 reflections
132 parameters
1 restraint
H-atom parameters constrained
Δρ_max_ = 0.15 e Å^−3^
Δρ_min_ = −0.14 e Å^−3^

### 

Data collection: *CrysAlis CCD* (Oxford Diffraction, 2006 ▶); cell refinement: *CrysAlis RED* (Oxford Diffraction, 2006 ▶); data reduction: *CrysAlis RED*; program(s) used to solve structure: *SIR97* (Altomare *et al.*, 1999 ▶); program(s) used to refine structure: *SHELXL97* (Sheldrick, 2008 ▶); molecular graphics: *ORTEP-3?* (Farrugia, 1997 ▶); software used to prepare material for publication: *WinGX* (Farrugia, 1999 ▶).

## Supplementary Material

Crystal structure: contains datablocks global, I. DOI: 10.1107/S1600536808023842/tk2285sup1.cif

Structure factors: contains datablocks I. DOI: 10.1107/S1600536808023842/tk2285Isup2.hkl

Additional supplementary materials:  crystallographic information; 3D view; checkCIF report

## Figures and Tables

**Table 1:** Hydrogen-bond geometry (Å, °)

*D*—H⋯*A*	*D*—H	H⋯*A*	*D*⋯*A*	*D*—H⋯*A*
N6—H6*B*⋯O1	0.89	2.05	2.890 (5)	157
N6—H6*A*⋯N5^i^	0.89	2.13	3.022 (4)	174
N7—H7*B*⋯N1^ii^	0.82	2.18	2.989 (4)	168
N7—H7*A*⋯N3^iii^	0.82	2.17	2.993 (4)	176

Symmetry codes: (i) 
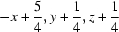
; (ii) 
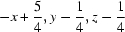
; (iii) 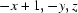.

**Fig. 1.**
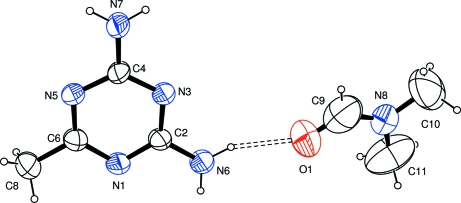
The molecular structure of (I), showing the atom-labelling scheme. Displacements ellipsoids are at the 50% probability level.

**Fig. 2.**
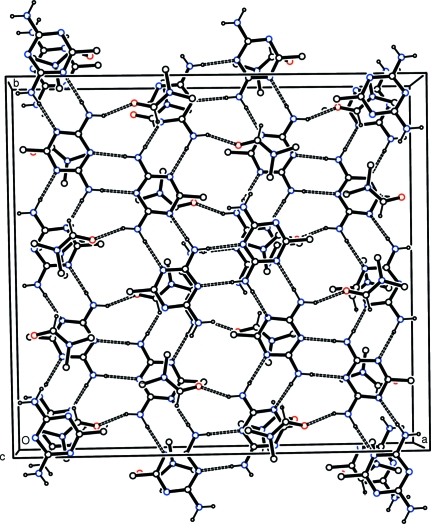
Crystal packing diagram for (I) viewed approximately down the c-axis. All atoms are shown as small spheres of arbitrary radii. For the sake of clarity, only H atoms involved in hydrogen bonding are shown. Hydrogen bonding is indicated by dashed lines.

## supplementary crystallographic information

### Comment

As a part of a more general study of multiple-hydrogen-bonding DNA/RNA nucleobases as potential supramolecular reagents (Portalone *et al.*, 1999; Portalone & Colapietro, 2007*a*, b), this work is a continuation of our studies on crystal adducts of DNA/RNA pyrimidine bases coupled with amino-derivatives of aromatic *N*-heterocycles *via* multiple hydrogen bonds to mimic the base-pairing of nucleic acids.

The asymmetric unit of (I) comprises a planar independent molecule of acetoguanamine hydrogen-bonded to *N*,*N*-dimethylformamide (DMF) (Fig. 1). A comparison of the molecular geometry of acetoguanamine with that reported for the corresponding molecule in the 1:1 monohydrated molecular adduct formed between acetoguanaminium chloride and acetoguanamine (Portalone & Colapietro, 2007*a*) shows that the corresponding bond lengths and angles are equal within experimental error. An analysis of the crystal packing of (I) shows (Table 1) that adjacent molecules of acetoguanamine are linked into ribbons (Fig. 2) by three independent intermolecular N—H···N hydrogen bonds between NH moieties and N atoms of the heterocyclic ring to form hydrogen-bonded rings (one centrosymmetric) of descriptor *R*^2^_2_(8) (Etter *et al.*, 1990; Bernstein *et al.*, 1995; Motherwell *et al.*, 1999). These hydrogen bonds that lead to two-dimensional arrays in the *ab* plane are bridged by DMF molecules *via* N–H ···O interactions forming C^1^_1_(3) chains.

### Experimental

Acetoguanamine (0.1 mmol, Sigma Aldrich at 98% purity) was dissolved in *N*,*N*-dimethylformamide (9 ml) and heated under reflux for 3 h. After cooling the solution to an ambient temperature, crystals suitable for single-crystal X-ray diffraction were grown by slow evaporation of the solvent after a few days.

### Refinement

All H atoms were found in a difference map, positioned with idealized geometry, and refined isotropically using a riding model (N–H = 0.82–0.89 Å, C–H = 0.93–0.97 Å). Their *U*_iso_ values were kept equal to 1.2*U*_eq_(N), 1.5*U*_eq_(C), 2.0*U*_eq_(C) of the solvent molecule. In the absence of significant anomalous scattering, Friedel pairs were merged.

### Crystal data

**Table:**

C_4_H_7_N_5_·C_3_H_7_NO	*F*_000_ = 1696
*M**_r_* = 198.24	*D*_x_ = 1.230 Mg m^−^^3^
Orthorhombic, *F**d**d*2	Mo *K*α radiation λ = 0.71073 Å
Hall symbol: F 2 -2d	Cell parameters from 11018 reflections
*a* = 25.548 (2) Å	θ = 3.0–25.6º
*b* = 23.0626 (19) Å	µ = 0.09 mm^−^^1^
*c* = 7.2689 (9) Å	*T* = 298 (2) K
*V* = 4282.8 (7) Å^3^	Tablets, colourless
*Z* = 16	0.15 × 0.14 × 0.14 mm

### Data collection

**Table:**

Oxford Diffraction Xcalibur S CCD diffractometer	1127 independent reflections
Radiation source: Enhance (Mo) X-ray source	698 reflections with *I* > 2σ(*I*)
Monochromator: graphite	*R*_int_ = 0.064
Detector resolution: 16.0696 pixels mm^-1^	θ_max_ = 25.9º
*T* = 298(2) K	θ_min_ = 3.0º
ω and φ scans	*h* = −31→31
Absorption correction: multi-scan(CrysAlis RED; Oxford Diffraction, 2006)	*k* = −27→28
*T*_min_ = 0.985, *T*_max_ = 0.990	*l* = −8→8
27177 measured reflections	

### Refinement

**Table:**

Refinement on *F*^2^	Secondary atom site location: difference Fourier map
Least-squares matrix: full	Hydrogen site location: inferred from neighbouring sites
*R*[*F*^2^ > 2σ(*F*^2^)] = 0.047	H-atom parameters constrained
*wR*(*F*^2^) = 0.127	*w* = 1/[σ^2^(*F*_o_^2^) + (0.0841*P*)^2^] where *P* = (*F*_o_^2^ + 2*F*_c_^2^)/3
*S* = 0.91	(Δ/σ)_max_ < 0.001
1127 reflections	Δρ_max_ = 0.15 e Å^−^^3^
132 parameters	Δρ_min_ = −0.14 e Å^−^^3^
1 restraint	Extinction correction: none
Primary atom site location: structure-invariant direct methods	

### Special details

**Table:**

Geometry. All e.s.d.'s (except the e.s.d. in the dihedral angle between two l.s. planes) are estimated using the full covariance matrix. The cell e.s.d.'s are taken into account individually in the estimation of e.s.d.'s in distances, angles and torsion angles; correlations between e.s.d.'s in cell parameters are only used when they are defined by crystal symmetry. An approximate (isotropic) treatment of cell e.s.d.'s is used for estimating e.s.d.'s involving l.s. planes.
Refinement. Refinement of *F*^2^ against ALL reflections. The weighted *R*-factor *wR* and goodness of fit *S* are based on *F*^2^, conventional *R*-factors *R* are based on *F*, with *F* set to zero for negative *F*^2^. The threshold expression of *F*^2^ > σ(*F*^2^) is used only for calculating *R*-factors(gt) *etc*. and is not relevant to the choice of reflections for refinement. *R*-factors based on *F*^2^ are statistically about twice as large as those based on *F*, and *R*- factors based on ALL data will be even larger.

### Fractional atomic coordinates and isotropic or equivalent isotropic displacement parameters (Å^2^)

**Table:**

	*x*	*y*	*z*	*U*_iso_*/*U*_eq_	
N1	0.63312 (11)	0.10945 (11)	0.5765 (4)	0.0495 (9)	
C2	0.58170 (12)	0.10315 (14)	0.6030 (6)	0.0431 (9)	
N3	0.55453 (10)	0.05414 (12)	0.5797 (4)	0.0447 (8)	
C4	0.58328 (13)	0.00862 (13)	0.5267 (5)	0.0416 (9)	
N5	0.63552 (11)	0.01049 (12)	0.4927 (5)	0.0471 (8)	
C6	0.65836 (13)	0.06118 (15)	0.5210 (5)	0.0463 (9)	
N6	0.55533 (11)	0.15017 (12)	0.6581 (5)	0.0592 (10)	
H6A	0.5721	0.1836	0.6753	0.071*	
H6B	0.5210	0.14808	0.6776	0.071*	
N7	0.55992 (11)	−0.04163 (11)	0.5010 (5)	0.0598 (10)	
H7A	0.5282	−0.04463	0.5175	0.072*	
H7B	0.5771	−0.0700	0.4684	0.072*	
C8	0.71557 (14)	0.06575 (18)	0.4923 (7)	0.0710 (13)	
H8A	0.7319	0.0329	0.5395	0.106*	
H8B	0.7282	0.0985	0.5522	0.106*	
H8C	0.7226	0.0687	0.3671	0.106*	
O1	0.44754 (15)	0.1783 (2)	0.7397 (7)	0.1196 (16)	
N8	0.36343 (14)	0.20435 (17)	0.7802 (5)	0.0738 (11)	
C9	0.4016 (3)	0.1667 (3)	0.7697 (9)	0.113 (2)	
H9	0.3931	0.1279	0.7864	0.226*	
C10	0.3107 (3)	0.1868 (4)	0.8156 (11)	0.160 (4)	
H10A	0.2905	0.1891	0.7028	0.319*	
H10B	0.2954	0.2122	0.9074	0.319*	
H10C	0.3105	0.1472	0.8606	0.319*	
C11	0.3722 (4)	0.2647 (2)	0.7568 (11)	0.140 (3)	
H11A	0.3839	0.2813	0.8722	0.279*	
H11B	0.3399	0.2833	0.7189	0.279*	
H11C	0.3988	0.2706	0.6634	0.279*	

### Atomic displacement parameters (Å^2^)

**Table:**

	*U*^11^	*U*^22^	*U*^33^	*U*^12^	*U*^13^	*U*^23^
N1	0.0426 (16)	0.0337 (16)	0.072 (2)	−0.0041 (13)	−0.0033 (15)	−0.0092 (16)
C2	0.0385 (18)	0.0307 (18)	0.060 (2)	−0.0004 (15)	−0.0048 (19)	−0.0032 (16)
N3	0.0391 (14)	0.0311 (14)	0.0638 (19)	−0.0001 (13)	−0.0004 (15)	−0.0059 (14)
C4	0.0400 (19)	0.0273 (17)	0.057 (2)	−0.0005 (14)	0.0046 (18)	−0.0045 (15)
N5	0.0406 (16)	0.0310 (15)	0.070 (2)	−0.0008 (12)	−0.0009 (16)	−0.0103 (15)
C6	0.0399 (17)	0.0381 (19)	0.061 (2)	−0.0037 (16)	−0.0027 (18)	−0.0038 (18)
N6	0.0457 (17)	0.0298 (15)	0.102 (3)	0.0009 (13)	−0.0020 (18)	−0.0156 (17)
N7	0.0414 (16)	0.0319 (15)	0.106 (3)	−0.0013 (13)	0.0115 (19)	−0.0126 (18)
C8	0.042 (2)	0.062 (2)	0.108 (4)	−0.0064 (19)	0.006 (3)	−0.019 (3)
O1	0.063 (2)	0.153 (4)	0.143 (4)	0.013 (2)	−0.005 (3)	−0.037 (3)
N8	0.070 (2)	0.072 (3)	0.079 (3)	0.006 (2)	0.0083 (19)	−0.012 (2)
C9	0.133 (6)	0.109 (5)	0.096 (5)	0.018 (5)	−0.008 (4)	−0.017 (4)
C10	0.101 (5)	0.266 (10)	0.112 (5)	−0.053 (6)	0.036 (4)	−0.050 (6)
C11	0.242 (9)	0.073 (4)	0.104 (5)	−0.002 (4)	0.001 (5)	0.008 (4)

### Geometric parameters (Å, °)

**Table:**

N1—C2	1.336 (4)	C8—H8B	0.9300
N1—C6	1.348 (5)	C8—H8C	0.9300
C2—N3	1.337 (4)	O1—C9	1.223 (7)
C2—N6	1.338 (4)	N8—C9	1.307 (7)
N3—C4	1.338 (4)	N8—C11	1.419 (6)
C4—N7	1.317 (4)	N8—C10	1.429 (7)
C4—N5	1.358 (4)	C9—H9	0.9300
N5—C6	1.323 (4)	C10—H10A	0.9700
C6—C8	1.480 (5)	C10—H10B	0.9700
N6—H6A	0.8907	C10—H10C	0.9700
N6—H6B	0.8907	C11—H11A	0.9700
N7—H7A	0.8226	C11—H11B	0.9700
N7—H7B	0.8226	C11—H11C	0.9700
C8—H8A	0.9300		
			
C2—N1—C6	115.1 (3)	C6—C8—H8C	109.5
N1—C2—N3	125.8 (3)	H8A—C8—H8C	109.5
N1—C2—N6	116.8 (3)	H8B—C8—H8C	109.5
N3—C2—N6	117.5 (3)	C9—N8—C11	121.7 (6)
C2—N3—C4	114.5 (3)	C9—N8—C10	121.7 (6)
N7—C4—N3	118.9 (3)	C11—N8—C10	116.6 (6)
N7—C4—N5	116.6 (3)	O1—C9—N8	125.6 (7)
N3—C4—N5	124.5 (3)	O1—C9—H9	117.2
C6—N5—C4	115.7 (3)	N8—C9—H9	117.2
N5—C6—N1	124.4 (3)	N8—C10—H10A	109.5
N5—C6—C8	118.5 (3)	N8—C10—H10B	109.5
N1—C6—C8	117.1 (3)	H10A—C10—H10B	109.5
C2—N6—H6A	120.0	N8—C10—H10C	109.5
C2—N6—H6B	120.0	H10A—C10—H10C	109.5
H6A—N6—H6B	120.0	H10B—C10—H10C	109.5
C4—N7—H7A	120.0	N8—C11—H11A	109.5
C4—N7—H7B	120.0	N8—C11—H11B	109.5
H7A—N7—H7B	120.0	H11A—C11—H11B	109.5
C6—C8—H8A	109.5	N8—C11—H11C	109.5
C6—C8—H8B	109.5	H11A—C11—H11C	109.5
H8A—C8—H8B	109.5	H11B—C11—H11C	109.5
			
C6—N1—C2—N3	0.2 (6)	N3—C4—N5—C6	2.1 (5)
C6—N1—C2—N6	180.0 (4)	C4—N5—C6—N1	−1.2 (6)
N1—C2—N3—C4	0.6 (6)	C4—N5—C6—C8	178.1 (4)
N6—C2—N3—C4	−179.2 (3)	C2—N1—C6—N5	0.2 (6)
C2—N3—C4—N7	179.7 (4)	C2—N1—C6—C8	−179.2 (4)
C2—N3—C4—N5	−1.8 (5)	C11—N8—C9—O1	0.0 (10)
N7—C4—N5—C6	−179.4 (4)	C10—N8—C9—O1	179.9 (6)

### Hydrogen-bond geometry (Å, °)

**Table:**

*D*—H···*A*	*D*—H	H···*A*	*D*···*A*	*D*—H···*A*
N6—H6B···O1	0.89	2.05	2.890 (5)	157
N6—H6A···N5^i^	0.89	2.13	3.022 (4)	174
N7—H7B···N1^ii^	0.82	2.18	2.989 (4)	168
N7—H7A···N3^iii^	0.82	2.17	2.993 (4)	176

Symmetry codes: (i) −*x*+5/4, *y*+1/4, *z*+1/4; (ii) −*x*+5/4, *y*−1/4, *z*−1/4; (iii) −*x*+1, −*y*, *z*.
